# Strategy for Identification of Phosphorylation Levels of Low Abundance Proteins *in Vivo* for Which Antibodies Are not Available

**DOI:** 10.3390/jcdd4040017

**Published:** 2017-10-08

**Authors:** Kozo Hayashi, Ryo Yamashita, Ritsuko Takami, Toshikatsu Matsui, Masamitsu Gotou, Tomoyuki Nishimoto, Hiroyuki Kobayashi

**Affiliations:** 1 Integrated Technology Research Laboratories, Pharmaceutical Research Division, Takeda Pharmaceutical Company Limited, Kanagawa 251-8555 Fujisawa, Japan; ryo.yamashita@takeda.com (R.Y.); ritsuko.takami@takeda.com (R.T.); gotomasa@dream.com (M.G.); hiroyuki.kobayashi@takeda.com (H.K.); 2 Cardiovascular and Metabolic Drug Discovery Unit, Pharmaceutical Research Division, Takeda Pharmaceutical Company Limited, Kanagawa 251-8555 Fujisawa, Japan; toshikatsu.matsui@takeda.com (T.M.); tomoyuki.nishimoto@takeda.com (T.N.)

**Keywords:** β1-adrenergic receptor, immuno-affinity purification, knock-in mice, phosphorylation

## Abstract

Protein function is mainly modulated by dynamic reversible or irreversible post-translational modifications. Among them, the identification of protein phosphorylation sites and changes in phosphorylation levels *in vivo* are of considerable interest for a better understanding of the protein function. Thus, effective strategies for the quantitative determination of phosphorylation degrees for low abundant proteins, for which antibodies are not available, are required in order to evaluate the functional regulation of proteins attributed to phosphorylation. In this study, we used the heart β1-adrenergic receptor (Adrb1) as a model protein and developed FLAG-Adrb1 knock-in mice, in which the FLAG tag was inserted at the N-terminus of Adrb1. The phosphorylation sites and levels of Adrb1 in the heart were elucidated by immuno-affinity purification followed by quantitative mass spectrometry analysis using ion intensity ratio of the phosphorylated peptide versus corresponding unphosphorylated peptide. The phosphorylation levels at Ser274 and Ser462 of Adrb1 were approximately 0.25 and 0.0023. This effective strategy should be useful for not only analyzing site-specific phosphorylation levels of target proteins, but also quantifying the expression levels of proteins of interest when appropriate antibodies are not available.

## 1. Introduction

Phosphorylation is the most extensively studied post-translational modification (PTM) regulating protein activity and plays a pivotal role in cellular signaling events and metabolic processes. In particular, G-protein coupled receptors (GPCRs) are phosphorylated at multiple sites, acting as a barcode for regulating signaling in a tissue-specific manner through a combination of phosphorylation sites [1,2]. GPCRs are also reportedly dysregulated in several pathologies, such as cardiovascular diseases and nervous system disorders [3,4,5], and are targeted for pharmacological therapies [6,7]. As for the study of GPCRs, protein antibodies are powerful tools for understanding GPCRs by allowing expression levels and phosphorylation states to be evaluated. However, the development of GPCR-specific antibodies is generally difficult due to their complex structural properties. Moreover, commercially available antibodies do not frequently work due to the low-specificity of antibodies and the low expression level of GPCRs *in vivo*. To overcome these difficulties, we have established a strategy to analyze protein phosphorylation levels at specific sites *in vivo* using the GPCR β1-adrenergic receptor (Adrb1) as a model protein for which all commercially available antibodies do not work.

Adrb1 is predominantly expressed in the heart and plays a critical role in the regulation of heart rate and the force of myocardial contraction [8]. It is activated by catecholamines followed by phosphorylation in cardiomyocytes. In our previous study, we clarified the phosphorylation residues present on Adrb1 in the mouse heart as Ser274 and Ser280 in the third intracellular loop and Ser412, Ser417, Ser450, Ser451 and Ser462 in the C-terminus by exploiting advanced phosphoproteomic technologies [9]. Although the phosphorylation of Ser274, Ser280, and Ser462 was determined to be agonist-dependent, the stoichiometry of the phosphorylation was not fully investigated as the primary focus of the research was on enriched phosphorylated peptides to clarify the mechanisms underlying functional regulation associated with Adrb1 phosphorylation *in vivo* rather than the expression level and the distribution of total protein.

To quantitatively understand phosphorylation, changes in protein expression and phosphorylation should be integrated. For this purpose, a simple strategy is to utilize the ratio of the ion intensity of the phosphorylated tryptic peptide versus the unphosphorylated counterparts by mass spectrometry (MS) analysis as in the case of Western blotting (WB) [10,11]. To assess the precise quantitative phosphorylation state of Adrb1 at the physiological condition *in vivo*, a knock-in (KI) mouse of Adrb1 fused with FLAG-tag (FLAG-Adrb1 KI mouse) would potentially be an effective tool. In a KI model, the target gene is inserted into a specific locus in the genome via homologous recombination resulting in the natural expression pattern and level [12]. The FLAG epitope fused in-frame of Adrb1 enables anti-FLAG immunohistochemistry and WB as well as immunoprecipitation (IP).

In this study, we demonstrated the effectiveness of KI mice for determining protein phosphorylation levels at specific sites for a low abundant protein of interest.

## 2. Materials and Methods

### 2.1. Ethics Statement

All animal experiments were approved by the Institutional Animal Care and Use Committee of the Shonan Research Center, Takeda Pharmaceutical Company Limited.

### 2.2. Generation of FLAG-β1AR KI Mice

The targeting vector was constructed by chimeric intron, signal sequence, and three times repeated FLAG epitope-tagged mouse *Adrb1* coding DNA sequence (CDS) between the 5′ and 3′ arm to replace native *Adrb1* CDS. Embryonic stem ES cells derived from C57BL/6J mice were electroporated with the targeting vector, and antibiotic-resistant clones were screened by allele quantitative PCR for correct homologous recombination and the resulting loss of one native allele. The three positive clones were microinjected into Jcl:ICR (Clea Japan Inc, Tokyo, Japan) blastocysts to generate chimeric mice. A male chimera mouse of one clone was selected to perform *in vitro* fertilization with C57BL/6 female mice to obtain heterozygous KI mice for subsequent studies. PCR genotyping was performed with TaqMan Fast Universal PCR Master Mix (Life technologies) and the following primers and MGB probes: *Adrb1* forward (5′-ACAACCACTGTGGACAGCGATT), *Adrb1* MGB probe (5′-CGGAGTCCAAGGTGTAGAG), and *Adrb1* UTR reverse (5′-TCCGTGCGCCCAGAGA) to detect only the wild-type (WT) allele. KI forward (5′-GACACACTCCTGCTATGGGTACTG), KI MGB probe (5′-TGGTGACGAATTCG), and KI reverse (5′-TCGTGATCTTTGTAGTCACCATCA) were used to detect the KI allele. Ngf forward (5′-TGCATAGCGTAATGTCCATGTTG), Ngf VIC probe (5′-ACGGTTCTGCCTGTACGCCGATCA), and Ngf reverse (5′-TCTCCTTCTGGGACATTGCTATC) were used for the internal standard.

### 2.3. Quantitative Reverse Transcription PCR

RNA was isolated from the frozen heart using ISOGEN (Nippon Gene, Tokyo, Japan). Total RNA (1 μg) was taken from each sample and subjected to reverse transcription using SuperScript III (Invitrogen, Carlsbad, CA, USA). Using synthesized cDNA, quantitative reverse transcription PCR was performed with TaqMan Fast Universal PCR Master Mix (Life technologies, Carlsbad, CA, USA) and the following primers and probes: TaqMan Gene Expression Assays Mm00431701 (Life technologies) to detect the *Adrb11* transcript derived from the native and KI allele, TaqMan Rodent GAPDH Control Reagents (Life technologies) for the internal standard, and KI forward (5′-GACACACTCCTGCTATGGGTACTG), KI MGB probe (5′-TGGTGACGAATTCG), and KI reverse (5′-TCGTGATCTTTGTAGTCACCATCA) to detect the Signal sequence of FLAG (Sig S-FLAG). The relative mRNA expression of *Adrb1* and Sig S-FLAG was determined using the ΔCt method (value obtained by subtracting the Ct value of *Gapdh* mRNA from that of the target mRNA). Data were expressed as the ratio (calculated using 2^−(ΔCt)^) of target mRNA to *Gapdh* mRNA.

### 2.4. Preparation of Heart Membrane Proteins 

Isolated mouse heart was cut into small pieces with scissors in ice cold homogenization buffer [20 mM Tris-HCl (pH 7.4)] containing phosphatase and protease inhibitor cocktails (Sigma-Aldrich, St. Louis, MO, USA) and homogenized using a physcotron homogenizer (Microtech Co., LTD, Chiba, Japan) for 30 s. The homogenate was centrifuged at a speed of 2000× *g* for 10 min, and the supernatant was ultracentrifuged at 200,000× *g* for 30 min. The pellet was resuspended in radio-immunoprecipitation assay (RIPA) buffer without detergent [50 mM Tris (pH 7.5), 150 mM NaCl, and phosphatase and protease inhibitor cocktails], sonicated, and added with NP-40 at a final concentration of 1%. The lysate was mixed by inverting the tube several times every 10 min for 30 min at room temperature (RT) and ultracentrifuged for 30 min at 200,000× *g*. The recovered proteins were then subjected to IP.

### 2.5. IP of FLAG-Adrb1

FLAG-Adrb1 was purified using an anti-FLAG M2 affinity gel (Sigma-Aldrich). Briefly, to the membrane protein lysate prepared from FLAG-Adrb1 KI mouse heart (800 µL, total proteins: 1 mg), 40 µL of M2 affinity gel equilibrated with lysis buffer was added and rotated for 16 h at 4 °C. The resin was washed three times with lysis buffer and eluted with 40 µL of 0.3% sodium dodecyl sulfate (SDS) and 40 µL of 0.15% SDS.

### 2.6. Enzymatic Digestion of Immunoprecipitates

Immunoprecipitates were diluted with Milli-Q water and added with 1 µL of 1.5 M Tris-HCl (pH 8.8) followed by reduction with 5 mM Tris (2-carboxyethyl)phosphine hydrochloride. The reaction mixture was digested with 0.5 µg of Lys-C (Wako, Osaka, Japan) for 2 h at RT, followed by 1 µg of trypsin (Promega, Tokyo, Japan) for 16 h at RT. Protein digests were alkylated with 20 mM iodoacetoamide and subjected to strong cation exchange (SCX) column chromatography.

### 2.7. SCX Fractionation

SCX chromatographic fractionation was performed using a Shimazu HPLC system with a polysulfoethyl A SCX column (2.1 × 35 mm, 5 μm, 300A, PolyLC, Rumsey Rd, Columbia). Phosphorylated peptides were loaded on the column equilibrated with 80% acetonitrile (ACN) containing 0.1% HCO_2_H (Solvent A). The peptides were eluted with 30% ACN containing 350 mM NH_4_HCO_2_ at pH 3 (Solvent B) using a gradient from 0% to 10% B for 20 min, 10% to 20% B for 10 min, 20% to 40% B for 5 min, and 40% to 80% B for 5 min, followed by 80% to 100% B for 1 min at a flow rate of 0.1 mL/min.

### 2.8. Liquid Chromatography-Tandem Mass Spectrometry Analysis

Fractionated peptides were analyzed by online nano liquid chromatography (LC) using an Easy nLC1000 System (Thermo Fisher Scientific, Waltham, MA, USA) coupled to a Fusion Orbitrap mass spectrometer (Thermo Fisher Scientific). Peptides were loaded onto a trap column (C18 Pepmap100, 3 μm, 0.075 × 20 mm, Thermo Fisher Scientific) and resolved on an analytical column (Reprosil-Pur C18AQ 3 μm 0.075 × 150 mm, Nikkyo Technos, Tokyo, Japan) at 300 nL/min over 45 min. The mass spectrometer was operated collecting MS spectra in the Orbitrap mass analyzer at a resolution of 120,000 and data-dependent higher-energy collisional dissociation (HCD) tandem mass spectrometry (MS/MS) spectra in the ion trap with normalized collision energy of 30%. Peak areas in the extracted ion chromatogram (XIC) were calculated using Xcalibur software (version 3.0, Thermo Fisher Scientific).

When analyzing SCX fractions containing tryptic peptides extending from 458 to 466 on Adrb1 (QGFSSESKV), the MS scan ranges were set between *m*/*z* 465 and 540. The resolution was set to 500,000, and MS/MS was performed in a data-dependent acquisition scheme by selecting the target precursor ions at *m*/*z* 484.748 and 524.721 corresponding to unphosphorylated (QGFSSESKV) and phosphorylated peptides (QGFSpSESKV), respectively.

### 2.9. Database Search

All MS raw files were processed to identify and quantify peptides with Proteome Discoverer 1.4 (Thermo Fisher Scientific) using Mascot (version 2.5, Matrix Science, Boston, MA, USA) against the Uniprot mouse protein database. The mass tolerance of the precursor and fragment were set to 10 ppm and 0.45 Da. Up to one missed trypsin cleavage was allowed.

For Mascot search, the following parameters were set: carbamidomethylation of cysteine was selected as a fixed modification; oxidation of methionine, and the phosphorylation of serine, threonine, and tyrosine were selected as variable modifications. The phosphorylation site localization of identified phosphorylated peptides was performed by phosphoRS algorithm 3.1 implemented in Proteome Discoverer. A site localization probability of at least 0.75 was used as the threshold. A false discovery rate of 0.01 was applied to peptide identification.

### 2.10. Statistical Analysis

Welch’s *t*-test was performed to compare the mRNA expression for m*Adrb1* or Signal sequence of FLAG (Sig S-Flag) between wilt type (WT) and KI mice. *p* < 0.05 was considered statistically significant.

## 3. Results

### 3.1. Generation of FLAG-β1AR KI Mice

**Figure 1:**
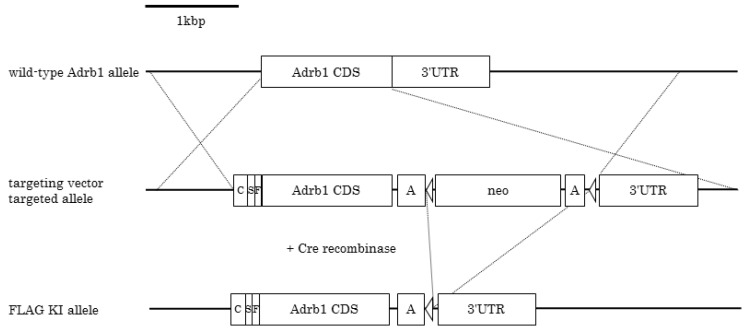
Targeted insertion of the gene for *Adrb1* in mice. Structure of the wild-type *Adrb1* allele (**top**), the targeting vector, and the targeted allele in mice ES cells (**middle**) and the resulting FLAG-tagged *Adrb1* KI mice after Cre recombination (**bottom**). C, chimeric intron and kozak sequence; S, signal sequence; F, three times repeat FLAG tag; A, polyadenylation sequence; neo, neo cassette; outlined triangle, loxP.

**Figure 2:**
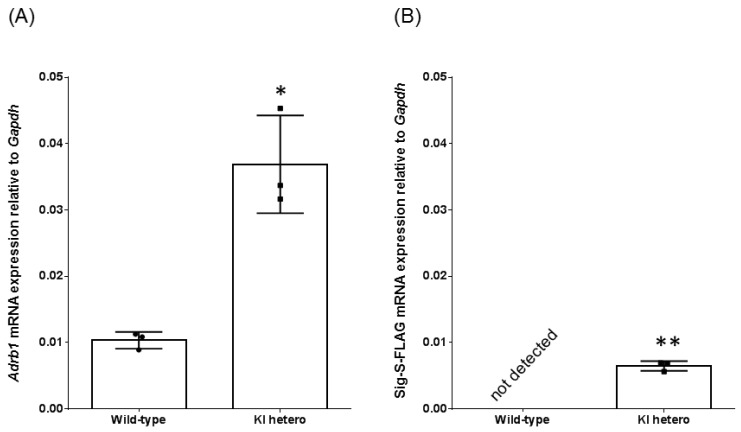
Quantitative analysis of *Adrb1* mRNA-expression (**A**) or Signal sequence of FLAG (Sig S-FLAG) (**B**) in hearts. Expression levels of *Adrb1* or Sig S-FLAG were determined by RT-qPCR and normalized to the housekeeping *Gapdh* gene. Data are expressed as the mean of three animals per group and expressed as mean ± SD WT, wild type; KI, knock-in mice. * *p* < 0.05 vs. WT (**A**), ** *p* < 0.01 vs. WT (**B**) (Welch’s *t*-test).

We targeted a construct containing the chimeric intron, signal sequence, and three times repeated FLAG epitope-tagged mouse *Adrb1* CDS into the *Adrb1* locus (Figure 1). To confirm the function of the KI allele, we performed quantitative PCR analysis of the heart. *Adrb1* transcript levels of WT mice and heterozygous mice were 0.010 and 0.037, respectively, and the levels of heterozygous mice were 3.6 times that of WT mice. The FLAG sequence-contained transcript was detected in only heterozygous KI mice as shown in Figure 2 (Supplementary Table S1).

### 3.2. Identification and Quantitative Analysis of the Phosphorylation of Adrb1-Derived Tryptic Peptides in the Heart of KI Mice

**Figure 3:**
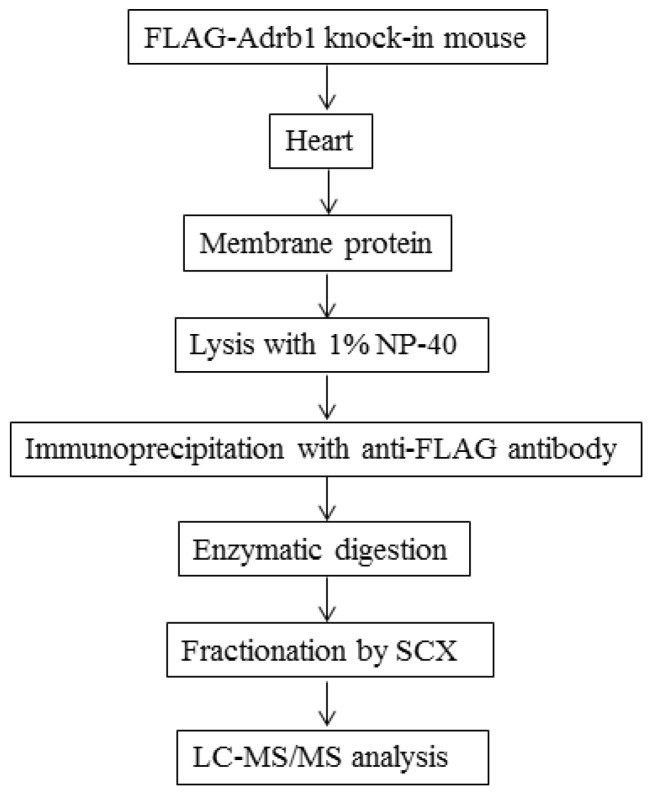
Flowchart describing the sequential steps for identifying β1AR-derived tryptic peptides in FLAG-Adrb1 knock-in (KI) mice. Membrane protein lysate prepared from the FLAG-Adrb1 KI mouse heart was immunoprecipitated with the anti-FLAG antibody. Immunoprecipitates were digested enzymatically and subjected to strong cation exchange (SCX) fractionation and LC–MS/MS analysis.

In our previous study, we identified the endogenous phosphorylation residues on Adrb1 *in vivo* by making full use of phosphoproteomic technologies [9]. However, the stoichiometry of the phosphorylation was not fully examined. To investigate the phosphorylation state of Adrb1 *in vivo*, the hearts of FLAG-Adrb1 KI mice were analyzed. The crude membrane protein prepared from the hearts was lysed with 1% NP-40, and the lysate was immunoprecipitated with the anti-FLAG antibody. Immunoprecipitates were enzymatically digested followed by reduction and alkylation. The resulting peptide mixture was fractionated by SCX column chromatography and analyzed by LC–MS/MS. Figure 3 shows the flowchart for identifying the tryptic peptides from Adrb1.

**Figure 4:**
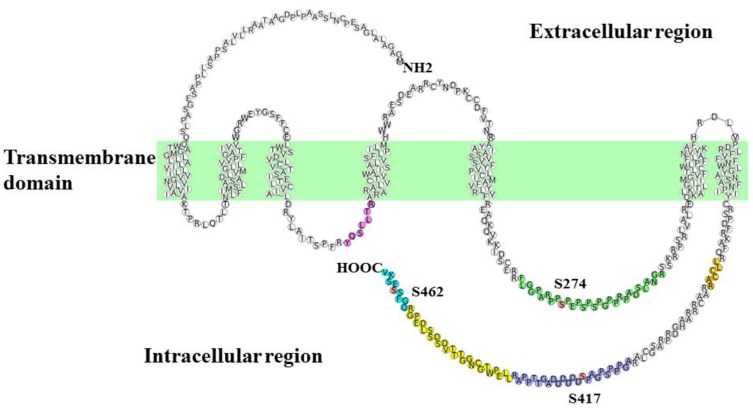
Schematic representation of mouse Adrb1 showing the location of identified tryptic peptide fragments. In total, six Adrb1-derived tryptic peptide fragments located in the third intracellular loop and the C-terminus of the receptor were identified. Each peptide fragment is shown as colored circles, with FLGGPARPPSPEPSPSPGPPRPADSLANGR as green, AGPPPSPGAPSDDDDDDAGTTPPAR as purple, LLEPWTGCNGGTTTV DSDSSLDEPGR as yellow, QGFSSESKV as cyan, LLCCAR as orange, and YQSLLTR as magenta. Phosphorylated residues are shown in red.

Interpretation of the HCD spectrum combined with a search against the UniProt mouse database using Mascot was utilized for the identification of each peptide sequence. Three Adrb1-derived tryptic phosphorylated peptides were identified together with their unphosphorylated counterparts. Moreover, three additional unphosphorylated peptide fragments at different sites of Adrb1 were detected as summarized in Table 1. The identified tryptic peptides were located in the second and the third intracellular loops and the C-terminus of Adrb1 as shown in Figure 4. 

To evaluate the phosphorylation level at each site, the ion intensities for the phosphorylated and the corresponding unphosphorylated peptides in MS were quantified by Xcalibur software, and the intensity ratios were calculated (Supplementary Table S2). The phosphorylation levels at Ser274 and Ser417 in the third loop of Adrb1 were high as compared with that at Ser462 in the C-terminus. 

## 4. Discussion

The process of activating GPCRs by agonist stimulation, followed by receptor internalization and trafficking events, is well documented; however, little of this process has been examined *in vivo*. To address this issue, a KI mouse can sometimes be a useful tool. Indeed, KI mice expressing a specific GPCR-fused green fluorescent protein have been produced to visualize the distribution and internalization of native GPCRs under agonist stimulation in an *in vivo* model [13,14,15,16]. Moreover, KI mice with biological function-impaired mutations have been generated to evaluate the physiological contribution of the target site by a gene KI approach [17,18,19].

In addition to these events, activities and interactions with other molecules of GPCRs are regulated by phosphorylation of the receptor. Therefore, direct analysis of the phosphorylation state *in vivo* is particularly important; however, at the same time, this is enormously difficult due to the low stoichiometry of phosphorylation events in natural states [20,21]. To overcome these difficulties, enrichment of phosphorylated peptides is effective. However, information on the expression level of proteins is lost in the course of enrichment. Thus, individual quantitative analysis of global protein expression and phosphorylation levels is required for a precise interpretation of phosphorylation changes; however, it is very challenging to detect an extremely low abundant unmodified peptide fragment with global proteomic analysis without appropriate antibodies. To analyze the phosphorylation state in a quantitative manner by MS technology, isotope dilution is a standard method [22,23]. Furthermore, multiple-reaction monitoring is a useful method for measuring the absolute quantity of phosphorylation occupancy at a specific site in a protein of interest [24]. However, these methods require the synthesis of isotope-labeled peptides, which is time and resource intensive, and in most cases, enrichment of phosphorylated peptides is also required for low abundant proteins.

In contrast, a simple method to assess the extent of phosphorylation is the use of the ion intensity ratio calculated from the phosphorylated and corresponding unphosphorylated tryptic peptides in MS analysis, since these are closely matched in terms of amino acid composition and retention time on HPLC. In general, it is well known that the ionization efficiency of peptide strongly depends on their modification state and the coeluting materials as well as amino acid composition. In particular, an addition of phosphate group gives the suppression of the ionization in MS. Although the ratio does not directly translate into stoichiometry, the change in ion intensity ratios between phosphorylated and corresponding unphosphorylated peptides is indicative of changes in the level of phosphorylation. 

Taking these points into consideration, a mouse model where a protein of interest is fused with an epitope tag such as FLAG or hemagglutinin is a powerful tool for analyzing the phosphorylation states of target proteins [25,26]. Therefore, in this study, we have generated a KI mouse expressing Adrb1 and engineered to contain an N-terminal FLAG epitope adequate for immuno-affinity purification. We believed that the small FLAG-tag would not disrupt the function of Adrb1 in the mice. The generated KI mice showed an elevation in the mRNA levels of *Adrb1* compared to WT mice despite the same copy number; however, the promoter region and 3' UTR of *Adrb1* were contained in the targeting construct of KI mice. Therefore, we consider that the localization of Adrb1 protein would be maintained in the KI mice. It is known that the expression levels were influenced by transcriptional regulatory elements of targeting constructs, such as promoter, intron and polyadenylation (polyA) signal sequence [27]. In the KI mice, chimeric intron or TK polyA sequence might affect the *Adrb1* mRNA levels.

To examine phosphorylation levels at specific sites on Adrb1 in KI mouse hearts, we made use of proteomic technology, which combined the specificity of antibody-based purification with the high sensitivity of MS analysis. As a result, six tryptic peptide fragments were identified, and three were detected together with their phosphorylated peptides from the membrane protein fraction in the KI mouse heart (Table 1). All the phosphorylated fragments identified in the FLAG-Adrb1 KI mouse by use of immune-affinity purification were consistent with those identified in isolated perfused normal mouse heart by TiO_2_ purification [9]. On the other hand, peptide fragments of Adrb1 could not be detected at all from the soluble protein fraction of the heart (data not shown). These indicate the adequate localization of FLAG-Adrb1 in the mouse heart. Furthermore, without the immune-affinity or TiO_2_ purification, the Adrb1 tryptic peptides could not be detected in an analysis of total proteins derived from a single intact heart. In particular, in the case of Adrb1, it was difficult to identify the specific band of FLAG-Adrb1 in WB analysis using anti-FLAG antibody even after immune-affinity purification (data not shown). On the contrary, the target peptide fragment was assigned with certainty in the MS analysis. 

As for the phosphorylation sites, phosphorylations at Ser274 and S462, which previously were not detected in the perfused mouse heart without the stimulation of ligand [9], were observed at the basal level in the Adrb1 KI model without agonist treatment even though the phosphorylation level at S462 was extremely low. This may be because the basal phosphorylation of these sites in the heart was reduced artificially in the perfusion medium, which did not contain catecholamines. On the other hand, the phosphorylation sites at Ser450 and Ser451, phosphorylated in the perfused mouse model, were not detected in the KI mouse model. We believe the analysis in KI mice would reflect the natural physiological phosphorylation state.

As for the quantitative analysis of phosphorylation, the phosphorylation level at each site was determined reproducibly except for Ser462 whose ion intensity was extremely low compared with the other sites. These data demonstrate that FLAG-Adrb1 KI mouse will be applicable for analyzing the stoichiometry of Adrb1 phosphorylation *in vivo*. Next to clarifying the relative stoichiometry of phosphorylated forms in Adrb1, the determination of the absolute phosphorylation site occupancy which determines the nature of the signaling response is an important aspect and the issue in the future. This KI mouse can also be utilized to analyze the expression of the receptor without radioligands by quantifying the unphosphorylated peptide in MS analysis. Furthermore, this mouse line may be an effective model for investigating the physiological functions of Adrb1 *in vivo*, such as distribution, trafficking and characterization. In conclusion, we have established an effective strategy for quantitatively analyzing protein phosphorylation levels at specific sites *in vivo*.

## Figures and Tables

**Table 1:** Tryptic peptide fragments and phosphorylation ratios in FLAG-Adrb1 KI mouse heart.

Sequence	Start	End	Phos-Site	*m*/*z*	Charge	Phosphorylation ratio ^a^ (Phospho/Nonphospho)
FLGGPARPPpSPEPSPSPGPPRPADSLANGR	265	294	S274	765.38	4	0.25 ± 0.058
AGPPPSPGAPpSDDDDDDAGTTPPAR	407	431	S417	819.34	3	0.56 ± 0.070
QGFSpSESKV	458	466	S462	524.72	2	0.0023 ^b^
YQSLLTR	166	172	-	440.75	2	-
LLCCAR	379	384	-	396.70	2	-
LLEPWTGCNGGTTTVDSDSSLDEPGR	432	457	-	922.09	3	-

The phosphorylation ratio Phospho/Nonphospho corresponds to the ratio of the peak intensities of the phosphorylated peptides versus corresponding unphosphorylated counterparts in extracted ion chromatogram.^a^ Each value shows the mean ± SD (*n* = 3). ^b^ Mean of two experiments.

## Supplementary Materials

The following are available online at www.mdpi.com/2308-3425/4/4/17/s1, Table S1: Raw data for quantitative reverse transcription PCR, Table S2: Raw data for tryptic peptide fragments and phosphorylation ratios in FLAG-Adrb1 KI mouse heart.
